# Zika virus prevention behaviors and knowledge among male partners of pregnant people and lack of condom use as a prevention behavior from the *Zika en Embarazadas y Niños* (ZEN) prospective cohort study, Colombia

**DOI:** 10.1186/s13104-024-06702-1

**Published:** 2024-03-21

**Authors:** Christina L. Sancken, Ayzsa Tannis, Sandra A. Amouzou, Veronica Burkel, Jeffrey M. Carlson, Suzanne Newton, Suzanne M. Gilboa, Maritza Gonzalez, Diana Valencia, Van T. Tong, Martha Ospina

**Affiliations:** 1 U.S. Centers for Disease Control and Prevention, Atlanta, GA USA; 2 Eagle Global Scientific, LLC, San Antonio, TX USA; 3 Rollins School of Public Health, Emory University, Atlanta, GA USA; 4 Instituto Nacional de Salud de Colombia, Bogotá, Colombia

**Keywords:** Zika virus, Knowledge, attitudes, behaviors, Colombia, Pregnancy, Prevention

## Abstract

**Objective:**

Zika virus (ZIKV) infection in pregnancy can cause brain and eye abnormalities and neurodevelopmental sequelae. In the absence of medical countermeasures, behavioral interventions were recommended to prevent mosquito bites and sexual transmission of ZIKV. This report uses data from the *Zika en Embarazadas y Niños* (ZEN) prospective cohort study in Colombia to describe the knowledge, attitudes, and behaviors (KAB) related to ZIKV prevention in male partners compared to those of their pregnant partners at study enrollment during February 2017–2018.

**Results:**

Most male partners reported wearing protective clothing such as long pants (97.6%) and long sleeves (72.8%), as well as covering ankles and feet (89.1%) to prevent ZIKV infection. When comparing the preventive behavior of condom use between male and pregnant partners, 26 pairs (10.0%) both responded that they performed the behavior. Overall, 25.1% of male partners and 18.9% of pregnant people reported any condom use during the three months before enrolling in ZEN. When comparing other preventive behaviors between male and pregnant partners, the behavior which was most frequently reported by both partners was wearing long pants (85.4%), and the least frequently reported by both partners was using condoms after finding out about a partner’s pregnancy (3.4%).

**Supplementary Information:**

The online version contains supplementary material available at 10.1186/s13104-024-06702-1.

## Introduction

Zika virus (ZIKV) transmission primarily occurs through the bite of an infected mosquito, in utero from mother to fetus, or through sexual transmission. The consequences of ZIKV infection during pregnancy can be severe, including congenital Zika syndrome and possible neurodevelopmental abnormalities, especially if the infection occurs in early pregnancy [1, 2].

The first ZIKV outbreak in Colombia was identified in October 2015, and by the end of July 2016, it was estimated that just over 18,000 pregnant people were affected by Zika Virus Disease, as reported to Colombia’s national surveillance system [3, 4]. During the height of the epidemic, the prevalence of brain or eye defects in Colombian infants or fetuses was 13 per 10,000; higher than pre- and post-epidemic estimates [4]. Although the end of the Zika epidemic was declared in July 2016 [5], Zika virus continued circulating at lower levels than during the epidemic.

From the beginning of the epidemic through 2016, 106,659 cases were reported to Colombia’s national surveillance system, with 19,746 reported among pregnant people [6]. In 2017, 1,901 cases were reported, with 265 reported among pregnant people, and 241 cases were reported from January–February 2018, with 61 reported among pregnant people [7, 8].

In 2017, Colombia’s *Instituto Nacional de Salud* (INS) and the U.S. Centers for Disease Control and Prevention (CDC) launched a prospective pregnancy cohort study *Zika en Embarazadas y Niños* (ZEN) in three departments of Colombia with high numbers of ZIKV cases reported during the ZIKV outbreak. Unique to this cohort, male partners were also enrolled. This report describes the knowledge, attitudes, and behaviors (KAB) around the prevention of sexual transmission of ZIKV among the male partners of pregnant people in areas where ZIKV was circulating. Because male partners infected with ZIKV can transmit the virus to their pregnant partners through sex, and because one partner’s attitudes toward prevention behaviors can shape the other partner’s actions, understanding male partners’ KAB can inform strategies to prevent ZIKV infection.

## Methods

Pregnant people in their first trimester were recruited from 13 public and private prenatal clinics based on study inclusion criteria [9]. At their study enrollment visit, pregnant people were asked if a male partner lived with them and, if so, whether study staff could contact him. Male partners aged 18 years and older were enrolled between February 2017 and February 2018 and visited the prenatal clinics to complete an interviewer-administered enrollment questionnaire. Questions included behaviors to prevent ZIKV infection performed in the past seven days: wearing long pants, wearing long sleeves, covering ankles and feet, using mosquito repellent; using condoms during sex, and if the frequency of condom use changed after finding out their partner was pregnant. Further details on the ZEN cohort methods and objectives are described elsewhere [9].

Analyses were conducted in SAS 9.4 (Cary, NC). With the exception of change in condom use, variables with more than two response options were dichotomized; for example, if responses could be ‘always’, ‘sometimes’, ‘never’, or ‘don’t know’, ‘always’ and ‘sometimes’ were grouped and ‘never’ and ‘don’t know’ were grouped. For the question about change in condom use after finding out a partner was pregnant, response options were dichotomized as ‘any change in condom use’ (where condom use was reported more often or where condom use was reported less often) and ‘no change in condom use’ (where no change in condom use, do not use condoms, or don’t know were reported). A sensitivity analysis was performed with ‘always’ and ‘sometimes’ grouped, and ‘never’ alone, excluding ‘don’t know’. Frequencies, number, and percent where both the male and pregnant partner reported engaging in a behavior, and simple Kappa coefficients that account for agreement among male and pregnant partners, excluding agreement by chance, were used to describe KAB among male partners and pregnant people enrolled in ZEN. Kappa coefficients were considered as: no agreement (≤ 0), none to slight agreement (0.01–0.20), fair agreement (0.21–0.40), moderate agreement (0.41–0.60), substantial agreement (0.61–0.80), and almost perfect agreement (0.81–1.0) [10].

## Results

Results on the enrollment of pregnant people and male partners, inclusion and exclusion criteria, and reasons for not participating are described elsewhere [9]. Data from 287 pregnant people and their male partners enrolled in ZEN were used in this analysis. Of 287 participating male partners, 56.1% were 28 years or older, 35.5% completed technical school or university, 67.6% had public or private health insurance compared to none or unknown insurance status, 98.3% were married or in a free union, and 79.1% had low socioeconomic status as defined by the Colombian government [11].

A higher proportion of males responded that someone in their community could be infected with ZIKV (75.4% vs. 67.6%), and when asked about the likelihood of a baby having intrauterine growth restriction (IUGR) if the pregnant person has ZIKV (81.8% vs. 73.7%) compared to their pregnant partners. Males and pregnant partners responded similarly in that they were very or somewhat worried about contracting ZIKV (84.4% vs. 89.9%), that it was very or somewhat likely that a baby could be born with microcephaly if the pregnant person has ZIKV (87.6% vs. 86.6%), that it was very or somewhat likely a baby could be born with other congenital anomalies if the pregnant person has ZIKV (87.2% vs. 83.0%), and that it was very or somewhat likely that a pregnant person with ZIKV could have a pregnancy loss or stillbirth (78.23% vs. 80.4%) (Table 1).

**Table 1:** ZIKV Knowledge, Attitudes, and Behaviors between Males and their Pregnant Partners Enrolled in ZEN

	Male Partners n (%)	Pregnant Partners n (%)	Both Reported ‘Yes’, ‘Very/Somewhat’, or Performed a Behavior n (%)	Simple Kappa Coefficient (95% confidence interval)
Knowledge and Attitudes				
Someone in community can be infected with Zika? (N = 284)				
Yes	214 (75.4)	192 (67.6)	144 (50.7)	− 0.0116 (− 0.1252, 0.1020)
No/Don’t Know	70 (24.7)	92 (32.4)		
Level of worry about getting Zika (N = 276)				
Very/Somewhat Worried	233 (84.4)	248 (89.9)	210 (76.1)	0.0205 (− 0.0997, 0.1407)
Not Worried/Don’t Know	43 (15.6)	28 (10.1)		
Likelihood a baby is born with microcephaly if mother infected with Zika? (N = 283)				
Very/Somewhat Likely	248 (87.6)	245 (86.6)	214 (75.6)	− 0.0220 (− 0.1317, 0.0877)
Not Likely/Don’t Know	35 (12.4)	38 (13.4)		
Likelihood a baby is born with other congenital anomalies if mother infected with Zika? (N = 282)				
Very/Somewhat Likely	246 (87.2)	234 (83.0)	205 (72.7)	0.0243 (− 0.0960, 0.1446)
Not Likely/Don’t Know	36 (12.8)	48 (17.0)		
Likelihood a baby has intrauterine growth restriction if mother infected with Zika? (N = 285)				
Very/Somewhat Likely	233 (81.8)	210 (73.7)	172 (60.4)	0.0063 (− 0.1073, 0.1199)
Not Likely/Don’t Know	52 (18.3)	75 (26.3)		
Likelihood of pregnancy loss/stillbirth if mother infected with Zika? (N = 285)				
Very/Somewhat Likely	223 (78.3)	229 (80.4)	181 (63.5)	0.0388 (− 0.0813, 0.1590)
Not Likely/Don’t Know	62 (21.8)	56 (19.7)		
Behaviors				
Long pants covering legs^a^ (N = 287)				
Always/Sometimes	280 (97.6)	252 (87.8)	245 (85.4)	− 0.0424 (-0.0693, − 0.0155)
Never	7 (2.4)	35 (12.2)		
Long sleeves covering arms^a^ (N = 287)				
Always/Sometimes	209 (72.8)	187 (65.2)	143 (49.8)	0.1104 (− 0.0063, 0.2270)
Never	78 (27.2)	100 (34.8)		
Feet and ankles covered^a^ (N = 285)				
Always/Sometimes	254 (89.1)	166 (58.3)	145 (50.9)	− 0.0474 (− 0.1267, 0.0318)
Never	31 (10.9)	119 (41.8)		
Mosquito repellent^a^ (N = 284)				
Always/Sometimes	53 (18.7)	50 (17.6)	17 (6.0)	0.1819 (0.0475, 0.3163)
Never	231 (81.3)	234 (82.4)		
Vaginal sex^b^ (N = 261)				
Did not abstain	258 (98.9)	257 (98.5)	256 (98.1)	0.5657 (0.1237, 1.0000)
Abstained	3 (1.2)	4 (1.5)		
Condom use^c^ (N = 259)				
Always/Sometimes	65 (25.1)	49 (18.9)	26 (10.0)	0.3065 (0.1731, 0.4399)
Never	194 (74.9)	210 (81.1)		
Condom use changed after finding out partner was pregnant ^d^ (N = 237)				
Any change in frequency of condom use	40 (16.9)	26 (11.0)	8 (3.4)	0.1262 (− 0.0227, 0.2751)
No change in frequency of condom use	197 (83.1)	211 (89.0)		

^a^ Responses indicate whether the person performed the behavior in the seven days prior to enrollment

^b^ Responses indicate whether the person abstained from vaginal sex in the three months prior to enrollment

^c^ Responses indicate whether the person used a condom during sex in the three months prior to enrollment

^d^ Where respondents reported always or sometimes using a condom with their partner in the three months prior to enrollment

Where both male and pregnant partners responded ‘yes’ or that it was ‘very or somewhat likely’ to the same knowledge or attitude question, the percentage was highest for being somewhat or very worried about contracting ZIKV (76.1%) and lowest for whether someone in the community could have ZIKV (50.7%) (Table 1). All Kappa coefficients fell in the no agreement or none to slight agreement categories, with the highest Kappa coefficient at 0.0388 for the question about the likelihood of pregnancy loss or stillbirth if the pregnant person has ZIKV.

Male partners were more likely than pregnant people to report engaging in Zika prevention behaviors in the last seven days, including wearing long pants (97.6% vs. 87.8%) and long sleeves that cover their arms (72.8% vs. 65.2%), covering their feet and ankles (89.1% vs. 58.3%), and using mosquito repellent (18.7% vs. 17.6%) (Table 1). In the three months prior to enrolling in ZEN, 25.1% of male partners reported condom use during sex, whereas 18.9% of pregnant people reported that their male partner used a condom. Of the male partners with reported condom use in the three months prior to enrolling in ZEN (25.1%), 82.6% answered the question around the frequency of use after finding out about their partner’s pregnancy, and 3.4% (8/237) reported any change in frequency of condom use. Of the pregnant people who reported that their male partner used a condom after finding out about the pregnancy, 11.0% (26/237) reported a change in the frequency of condom use during sex.

The percentage of couples where both male partners and pregnant partners reported engaging in a behavior to prevent ZIKV was highest for wearing long pants that cover legs (85.4%) and lowest for change in condom use after finding out about a partner’s pregnancy (3.4%) (Table 1). Kappa coefficients ranged from no agreement to moderate agreement, with the highest Kappa coefficient at 0.5657 for not abstaining from vaginal sex in the three months prior to enrolling in the study and the lowest at − 0.0474 for covering feet and ankles to prevent mosquito bites in the seven days prior to enrolling in ZEN.

Ten percent of males and pregnant partners both responded ‘always/sometimes’ to using condoms in the three months prior to enrollment (Table 1). Condom use after finding out a partner was pregnant changed in eight couples where each partner responded to this question (Table 1). While 74.9% of male partners reported no condom use, 18.6% of male partners reported condom use when pregnant partners indicated no condom use, and 11.9% of pregnant partners reported condom use when male partners did not indicate condom use (Additional file 1: Table S1). The Kappa coefficient for using condoms was considered to have a fair agreement, at 0.3065.

Results were not meaningfully different in the sensitivity analysis where ‘don’t know’ was excluded from the Kappa coefficients (Additional file 1: Table S2).

## Discussion and conclusions

Despite Colombian Ministry of Health and Social Protection and World Health Organization recommendations to prevent ZIKV in areas with high transmission rates [12, 13], ZIKV prevention behaviors were inconsistent between pregnant people and their male partners in this cohort study. Cultural norms or routine practice may play a role in behaviors that prevent or facilitate ZIKV infection [14–17].

Condom use was low among pregnant people and their male partners (10.0%) in this cohort study. Promoting condom use to prevent ZIKV transmission while considering gender norms and cultural context is a complex issue. In qualitative studies on gender and ZIKV prevention behaviors in Latin America and the Caribbean, women are perceived as the home’s caretakers while men are perceived as the breadwinners and protectors of their families [14, 17]. Condom use or abstinence in a relationship lead to suspicion of infidelity and lack of trust [14, 17]. Considering condom use as a prevention measure for ZIKV and other arboviruses from both men’s and women’s perspectives in culturally relevant ZIKV and arbovirus prevention messaging may help increase the frequency of ZIKV preventive behaviors. For example, messaging that emphasizes condom use as a means of protecting one’s family from ZIKV and its impacts could appeal to men and may help strategic communications campaigns and other public health prevention activities focusing on men.

These findings provide an understanding of the knowledge, attitudes, and preventive behaviors male partners had concerning ZIKV after the large outbreak in Colombia. Comparing responses to questions around condom use between male partners and pregnant people can provide information for public health practitioners about the extent of condom use as a ZIKV prevention behavior in the population. Although there was fair to moderate agreement for some behavior-related questions, male partner and pregnant person responses to behavior-related questions were inconsistent. It is important to identify male partners’ opinions and perceptions so that challenges in recruiting male partners to participate in health-related studies can be understood and improved upon. The results from this study can be used to inform future public health campaigns to expand knowledge about and methods to prevent sexual transmission of ZIKV. Culturally appropriate campaigns could focus on including the roles that both men and pregnant people have in preventing the sexual transmission of ZIKV.

## Limitations

This analysis is subject to several limitations. First, these data are self-reported, and participants received information about the consequences of ZIKV infection during the enrollment process, potentially introducing social desirability bias, with participants more likely to report preventive behaviors. Second, only 20.5% (287/1,399) of eligible male partners agreed to participate. There are observed differences in characteristics between pregnant people whose male partner enrolled in ZEN and those who did not enroll; those who enrolled had a higher level of education, were more likely to have private insurance, and be married [9]. Third, condom use questions did not specifically ask about sex with the pregnant partner; some men reported having vaginal sex with more than one partner in the three months prior to enrolling in the study, meaning reported condom use may have been to prevent another sexually transmitted infection or to prevent pregnancy in a different partner and not necessarily to prevent ZIKV infection in the pregnant person. In this instance, the pregnant person would likely not supply a reliable response to the question. Additionally, condom use may have been low among males and their partners due to a perceived low risk of contracting and sexually transmitting ZIKV or because they were actively trying to get pregnant, despite public health recommendations to delay pregnancy during the ZIKV epidemic. Fourth, as participants were asked about their condom use in the three months before enrolling in ZEN, the time of conception overlapped with the time period condom use was assessed in the majority of pregnancies. This study did not assess partner behaviors at follow-up visits in relation to reduced burden of ZIKV transmission. Because this cohort was enrolled after the ZIKV epidemic ended in Colombia, the pattern and frequency of preventive behaviors may not represent the same level of perceived risk that people may have felt during the epidemic. Finally, as there is no comparable comparison group, we cannot be certain whether male partners or pregnant people changed ZIKV prevention behaviors due to pregnancy.

### Supplementary Information

**Additional file 1: Table S1.** Reported condom use between males and pregnant partners. **Table S2.** Sensitivity analysis of ZIKV knowledge, attitudes, and behaviors between males and their pregnant partners enrolled in ZEN.

## Data Availability

The datasets generated and analyzed during the current study are available from the corresponding author upon reasonable request.

## Abbreviations

CDC: U.S. Centers for Disease Control and Prevention
INS: Instituto Nacional de Salud (National Institute of Health)
IUGR: Intrauterine growth restriction
KAB: Knowledge, Attitudes, Behaviors
ZEN: Zika en Embarazadas y Niños (Zika in Pregnant People and Children)
ZIKV: Zika virus

## References

[1] Cuevas EL, Tong VT, Rozo N, Valencia D, Pacheco O, Gilboa SM (2016). Preliminary report of microcephaly potentially associated with Zika virus infection during pregnancy—Colombia, January-November 2016 MMWR. Morb Mortal Wkly Rep.

[2] Moore CA, Staples JE, Dobyns WB, Pessoa A, Ventura CV, Fonseca EB (2017). Characterizing the pattern of anomalies in congenital Zika syndrome for pediatric clinicians. JAMA Pediatr.

[3] Pacheco O, Beltrán M, Nelson C, Valencia D, Tolosa N, Farr SL (2020). Zika virus disease in Colombia—preliminary report. N Engl J Med.

[4] Instituto Nacional de Salud (INS). Boletín epidemiológico semanal: Semana epidemiológica número 30 de 2016 24 julio–30 julio. https://www.ins.gov.co/buscador-eventos/BoletinEpidemiologico/2016%20Boletin%20epidemiologico%20semana%2030.pdf. Accessed 22 Sep 2023.

[5] Ospina ML, Tong VT, Gonzalez M, Valencia D, Mercado M, Gilboa SM (2020). Zika virus disease and pregnancy outcomes in Colombia. N Engl J Med.

[6] Instituto Nacional de Salud (INS). Boletín epidemiológico semanal: Semana epidemiológica número 52 de 2016 25 diciembre–31 diciembre. https://www.ins.gov.co/buscador-eventos/BoletinEpidemiologico/2016%20Bolet%C3%ADn%20epidemiol%C3%B3gico%20semana%2052%20-.pdf. Accessed 11 Jan 2024.

[7] Instituto Nacional de Salud (INS). Boletín epidemiológico semanal: Semana epidemiológica 52–Dic 24 al 30 de 2017. https://www.ins.gov.co/buscador-eventos/BoletinEpidemiologico/2017%20Bolet%C3%ADn%20epidemiol%C3%B3gico%20semana%2052.pdf. Accessed 11 Jan 2024.

[8] Instituto Nacional de Salud (INS). Boletín epidemiológico semanal: Semana epidemiológica 09 Febrero 25 al 03 de marzo 2018. https://www.ins.gov.co/buscador-eventos/BoletinEpidemiologico/2018%20Bolet%C3%ADn%20epidemiol%C3%B3gico%20semana%2009.pdf. Accessed 11 Jan 2024.

[9] Gonzalez M, Tong VT, Rodriguez H, Valencia D, Acosta J, Honein MA (2020). Cohort profile: congenital Zika virus infection and child neurodevelopmental outcomes in the ZEN cohort study in Colombia. Epidemiol Health.

[10] McHugh ML (2012). Interrater reliability: the kappa statistic. Biochem Med.

[11] Departamento Administrativo Nacional de Estadística (DANE). Estratificación socioeconómica para servicios públicos domiciliarios. https://www.dane.gov.co/index.php/servicios-al-ciudadano/servicios-informacion/estratificacion-socioeconomica. Accessed 19 Dec 2023.

[12] Ministerio de Salud y Protección Social. Gestantes con Zika deben catalogarse como embarazos de alto riesgo. https://www.minsalud.gov.co/Paginas/Gestantes-con-zika-deben-catalogarse-como-embarazos-de-alto-riesgo-.aspx. Accessed 19 Decr 2022.

[13] World Health Organization (WHO). Vector control operations framework for Zika virus. https://www.who.int/publications/i/item/WHO-ZIKV-VC-16.4. Accessed 19 Dec 2022.

[14] Coutinho RZ, Montalvo AV, Weitzman A, Marteleto LJ (2021). Zika virus public health crisis and the perpetuation of gender inequality in Brazil. Reprod Health.

[15] Gurman T, Ballard A, Villanueva SF, Luis LD, Hunter G, Maloney S (2020). The role of gender in Zika prevention behaviors in the Dominican Republic: findings and programmatic implications from a qualitative study. PLoS Negl Trop Dis.

[16] Mendoza C, Jaramillo G-I, Ant TH, Power GM, Jones RT, Quintero J (2020). An investigation into the knowledge, perceptions and role of personal protective technologies in Zika prevention in Colombia. PLoS Negl Trop Dis.

[17] Bancroft D, Power GM, Jones RT, Massad E, Bernstein Iriart J, Preet R (2022). Vector control strategies in Brazil: a qualitative investigation into community knowledge, attitudes and perceptions following the 2015–2016 Zika virus epidemic. BMJ Open.

