# Supplementary material for: Zika virus prevention behaviors and knowledge among male partners of pregnant people and lack of condom use as a prevention behavior from the Zika en Embarazadas y Niños (ZEN) prospective cohort study, Colombia
Source: BMC Res Notes. 2024 Mar 21;17:87. doi: 10.1186/s13104-024-06702-1 (PMC10956169; doi:10.1186/s13104-024-06702-1)
Supplement: Supplementary file 1 — Additional file 1: Table S1. Reported condom use between males and pregnant partners. Table S2. Sensitivity analysis of ZIKV knowledge, attitudes, and behaviors between males and their pregnant partners enrolled in ZEN. [file 13104_2024_6702_MOESM1_ESM.docx]

# Additional File. Data Not Shown

## Table A1. Reported Condom Use Between Males and Pregnant Partners

|  | **Pregnant Partners** | |  |
| --- | --- | --- | --- |
| **Male Partners** | No Condom Use Reported  N (%) | Condom Use Reported  N (%) | Total (%) |
| No Condom  Use Reported  N (%) | 171 (88.1^; 81.4*) | 23 (46.9^; 11.9*) | 194 (74.9) |
| Condom Use  Reported  N (%) | 39 (18.6^; 60.0*) | 26 (53.1^; 40.0*) | 65 (25.1) |
| Total (%) | 210 (81.1) | 49 (18.9) | 259 (100) |

^Column percent

*Row percent

## Table A2. Sensitivity Analysis of ZIKV Knowledge, Attitudes, and Behaviors between Males and their Pregnant Partners Enrolled in ZEN

|  | Male Partners  n (%) | Pregnant Partners  n (%) | Both Reported ‘Yes’, ‘Very/Somewhat’, or Performed a Behavior  n (%) | Simple Kappa Coefficient  (95% confidence interval) |
| --- | --- | --- | --- | --- |
| **Knowledge and Attitudes** | | | | |
| Someone in community can be infected with Zika? (N=226) | | | | |
| Yes | 187 (82.7) | 187 (82.7) | 144 (63.7) | -0.0454  (-0.1665, 0.0757) |
| No/Don’t Know | 39 (17.3) | 39 (17.3) |  |  |
| Level of worry about getting Zika (N=276) | | | | |
| Very/Somewhat  Worried | 233 (84.4) | 248 (89.9) | 210 (76.1) | 0.0205  (-0.0997, 0.1407) |
| Not Worried/Don’t  Know | 43 (15.6) | 28 (10.1) |  |  |
| Likelihood a baby is born with microcephaly if mother infected with Zika? (N=231) | | | | |
| Very/Somewhat  Likely | 221 (95.7) | 224 (97.0) | 214 (92.6) | -0.0370  (-0.0557,  -0.0183) |
| Not Likely/Don’t  Know | 10 (4.3) | 7 (3.0) |  |  |
| Likelihood a baby is born with other congenital anomalies if mother infected with Zika? (N=224) | | | | |
| Very/Somewhat  Likely | 214 (95.5) | 215 (96.0) | 205 (91.5) | -0.0442  (-0.0641  -0.0242) |
| Not Likely/Don’t  Know | 10 (4.5) | 9 (4.0) |  |  |
| Likelihood a baby has intrauterine growth restriction if mother infected with Zika? (N=186) | | | | |
| Very/Somewhat  Likely | 175 (94.1) | 183 (98.4) | 172 (92.5) | -0.0260  (-0.0497,  -0.0024) |
| Not Likely/Don’t  Know | 11 (5.9) | 3 (1.6) |  |  |
| Likelihood of pregnancy loss/stillbirth if mother infected with Zika? (N=201) | | | | |
| Very/Somewhat  Likely | 188 (93.5) | 193 (96.0) | 181 (90.1) | 0.0485  (-0.1297, 0.2264) |
| Not Likely/Don’t  Know | 13 (6.5) | 8 (4.0) |  |  |
| **Behaviors** | | | | |
| Long pants covering legs ^a^ (N=287) | | | | |
| Always/Sometimes | 280 (97.6) | 252 (87.8) | 245 (85.4) | -0.0424  (-0.0693,  -0.0155) |
| Never | 7 (2.4) | 35 (12.2) |  |  |
| Long sleeves covering arms ^a^ (N=287) | | | | |
| Always/Sometimes | 209 (72.8) | 187 (65.2) | 143 (49.8) | 0.1104  (-0.0063, 0.2270) |
| Never | 78 (27.2) | 100 (34.8) |  |  |
| Feet and ankles covered ^a^ (N=285) | | | | |
| Always/Sometimes | 254 (89.1) | 166 (58.3) | 145 (50.9) | -0.0474  (-0.1267, 0.0318) |
| Never | 31 (10.9) | 119 (41.8) |  |  |
| Mosquito repellent ^a^ (N=284) | | | | |
| Always/Sometimes | 53 (18.7) | 50 (17.6) | 17 (6.0) | 0.1819  (0.0475, 0.3163) |
| Never | 231 (81.3) | 234 (82.4) |  |  |
| Vaginal sex ^b^ (N=261) | | | | |
| Did not abstain | 258 (98.9) | 257 (98.5) | 256 (98.1) | 0.5657  (0.1237, 1.0000) |
| Abstained | 3 (1.2) | 4 (1.5) |  |  |
| Condom use ^c^ (N=259) | | | | |
| Always/Sometimes | 65 (25.1) | 49 (18.9) | 26 (10.0) | 0.3065  (0.1731, 0.4399) |
| Never | 194 (74.9) | 210 (81.1) |  |  |
| Condom use changed after finding out partner was pregnant ^d^ (N=13) | | | | |
| Any change in  frequency of  condom use | 9 (69.2) | 10 (76.9) | 6 (46.2) | -0.3582  (-0.6243,  -0.0922) |
| No change in  frequency of  condom use | 4 (30.8) | 3 (23.1) |  |  |

^a^ Responses indicate whether the person performed the behavior in the seven days prior to enrollment.

^b^ Responses indicate whether the person abstained from vaginal sex in the three months prior to enrollment.

^c^ Responses indicate whether the person used a condom during sex in the three months prior to enrollment.

^d^ Where respondents reported always or sometimes using a condom with their partner in the three months prior to enrollment.
